# Associations Between Dietary Antioxidant Intake and Markers of Atherosclerosis in Middle–Aged Women From North-Western Algeria

**DOI:** 10.3389/fnut.2018.00029

**Published:** 2018-04-24

**Authors:** Mustapha Diaf, Meghit Boumediene Khaled

**Affiliations:** Department of Biology, Faculty of Natural and Life Sciences, Djillali Liabes University of Sidi-Bel-Abbes, Sidi Bel Abbes, Algeria

**Keywords:** atherosclerosis, dietary antioxidant, type 2 diabetes, middle-aged women, Algeria

## Abstract

**Background:** The role of several dietary antioxidants in preventing the development and the progression of atherosclerosis has recently aroused considerable interest. Although they are not yet conclusive, most of the existing suggestions support this hypothesis.

**Objective:** The aim of the present work was to investigate the intake of dietary antioxidant nutrients in relation to atherogenic indices in a group of Algerian middle aged women with and without type 2 diabetes.

**Methods:** A cross-sectional study was conducted on a group of middle-aged women from the north western region of Algeria. Anthropometric and biochemical parameters were measured. Dietary intake was assessed using a validated 3-days food record. Atherogenic indices -total cholesterol-to-high-density lipoprotein cholesterol ratio (TC/HDL) and apolipoprotein (apo) B-to-apo A1 ratio, were calculated. Associations between antioxidants dietary intake and atherogenic indices were examined using logistic regressions.

**Results:** 95 women with type 2 diabetes were compared to 93 non-diabetic ones. Statistical differences (*p* < 0.05) were revealed for body weight, height, body mass index (BMI), glycosylated hemoglobin (HbA1c) and total cholesterol levels. Furthermore, significant differences were noted for vitamin C, E and copper dietary intakes. The TC/HDL ratio was significantly associated to the highest quartiles of vitamin C in all patients; 3.519[2.405–4.408], *p* = 0.009 and in non-diabetic women; 3.984[1.775–7.412], *p* = 0.020, respectively. The odd ratios of vitamin E intakes were about 2.425[2.017–5.715], *p* = 0.012 in all patients and 1.843[1.877–2.731], *p* = 0.019 in non-diabetic group, respectively. However, the Apo B/Apo A1 ratio was more correlated to the highest quartiles of zinc and copper in non-diabetic group; OR = 0.059[0.006–0.572], *p* = 0.015 and 0.192[0.048–0.766], *p* = 0.019, respectively.

**Conclusion:** The estimated risk of atherosclerosis measured through the TC/HDL ratio was correlated to vitamins antioxidant intake, while the probable risk assessed by the Apo B/Apo A1 ratio was more associated to the mineral profile.

## Introduction

Diabetes mellitus constitutes a major risk factor of cardiovascular diseases (CVD), which in turn, are considered the most important cause of death in diabetic population [1].

Atherosclerosis, as a main cause of cardiovascular events, has an over tenfold risk when associated to diabetes and metabolic syndrome [2]. Recently, the role of antioxidants compounds in inhibiting atherosclerosis development is receiving an increasing attention from researchers and scientists [3].

The prevention of early atherogenic lesions, according to the oxidation hypothesis of atherosclerosis, is founded on the antioxidant defense system including both endogenously and exogenously (diet) derived compounds [4]. Likewise, reducing the atherogenic risk by protecting low density lipoprotein (LDL) particles from oxidation is being increasingly discussed. Dietary micronutrients like vitamin C (ascorbic acid), vitamin E (e.g., α-tocopherol), β-carotene (provitamin A), and zinc (Zn) are known for their antioxidant properties and have received the greatest attention with regard to coronary heart disease prevention. Dietary supplements of vitamin E, vitamin C, β-carotene, or a combination of antioxidants were shown to inhibit lipid peroxidation *ex vivo* [5]. Other micronutrients (e.g., magnesium (Mg), and copper (Cu)) are known for their essential roles in enzymatic functions [6, 7]. However, despite the probable involvement of dietary components in the occurrence of various CVD; there are no definite dietary guidelines for preventing the occurrence of atherosclerosis [8].

Scarce studies examined the relationship between antioxidant vitamin status and earlier stages of atherosclerosis. Similarly, the protective effect of dietary antioxidants against cardiovascular risk factors has not been entirely enlightened with regard to pathophysiological features. The beneficial antioxidant effects of fruit and vegetable intake are being studied in several diseases, especially cardiovascular events [9, 10]. However, the evidences that fruit and vegetable consumption prevents atherosclerotic event remains limited [8].

Recently, cumulative suggestions are supporting the pivotal role of lipoproteins [total cholesterol to high density lipoprotein (TC/HDL)] and apolipoproteins (Apo B/Apo A1) ratios as consistent predictors of atherogenic risk, at an earlier stage, than lipids alone [11–15].

This study aims to evaluate the relationship between dietary intake of antioxidant vitamins (A, C, and E) and minerals (Mg, Zn, and Cu) and two lipid ratios that are considered as potential metabolic markers of atherosclerosis (TC/HDL and Apo B/Apo A1 ratios) in a group of middle-aged women. The main study population was composed of type 2 diabetic and non-diabetic women from the north western region of Algeria.

## Patients and methods

### Study population

A cross-sectional study was carried out during 5 months, from March till July 2015, and included 188 women (95 were type 2 diabetic and 93 were non-diabetic), free of clinical CVD at baseline recruited at the level of three health facilities located in the north-western region of Algeria; diabetes center “*Maison du diabétique*” and Mostefa Ben Brahim polyclinic, in Sidi-Bel-Abbes city, and Meslem Tayeb Hospital, in Mascara city.

Subjects who do not met the exclusion criteria (pregnant women, those under insulin therapy or with hypothyroidism, patients with primary hyperlipidaemia, renal impairment, and liver dysfunction) were solicited to participate in our study. On the base of a careful analysis of their medical records, patients' selection was performed during the medical follow-up sessions. The simple random sampling method without replacement was used. During the study period each women (coming for medical consultation or medical follow-up during the study period) has the same opportunity to participate in our study.

The control group was selected according to the same considerations except that these women were not diabetics.

The sample size was calculated using the following equation

$$\begin{array}{l} n_{0}=\left[{\text{Z}}^{2}*\text{P}\left(1-\text{P}\right)\right]/{\text{e}}^{2} \end{array}$$

(*Z*) is the level of confidence (1.96 corresponding at 95%), (*P*) is the initial level of the indicators is the true value of the percentage of favorable patients (type 2 diabetic patients responding to inclusion and exclusion criteria; according to our methods and patients session) and (*e*) is the margin of error. After that the following equation was employed;

$$\begin{array}{l} n=\frac{n_{0}}{1+\frac{n_{0}}{N}} \end{array}$$

(n_0_) is the sample size (without correction), (N) is the population size and (n) is the sample size corrected (final sample).

The number of type 2 diabetes patients in Sidi-bel-Abbes and Mascara cities was about 22,000. About 59% (nearly 10,980 patients) were females and about 12% (nearly 1,262 patients) were treated solely with oral anti-diabetic agents and responding to our inclosing and exclusion criteria.

The estimated sample of patients who were supportive to be part of our study was about (n_0_ ≈ 101) (type 2 diabetes patients). After correction using the formula:

$$\begin{array}{l} n=\frac{n_{0}}{1+\frac{n_{0}}{N}} \end{array}$$

The sample was approximately about (n ≈ 99). In our study, four patients did not finish all the steps of the investigation and their results were incomplete (removed from the study)

Regarding the control group, patients were selected according to the same considerations except that these women were not diabetics.

### Anthropometric measurements

The measurements of anthropometric dimensions were performed in the morning according to the World Health Organization (WHO) recommendations' [16]. Respecting the appropriate position for stature measurement (gathered feet, straight body, heels touching the wall, and staring out the horizon), and for the body weight evaluation (all participants were asked to be lightly dressed during the weight evaluation in order to minimize the weight of clothing). The body height (in meters) was measured with a body meter (Seca 206, Germany; measuring range 0–220 cm, graduation length 1 mm). Body weight was recorded using an electronic scale (TS-2003A: 360 lb, Capacity: 180 kg, Graduations 0.1 kg). Then, the Body Mass Index (BMI) was calculated as weight (kg)/height^2^ (m^2^).

The waist circumference was obtained using a measuring tape (maximum 150 cm, graduation length 1 mm). The tape was gently tightened around the patient's abdomen in the line passing bit above the navel just above the uppermost lateral border of the ilium.

### Blood pressure measurement

The Blood Pressure Monitor OMRON *M3* (Omron Healthcare., Ltd. Kyoto, Japan) was employed for measuring blood pressure in the morning. The patients were in a semi-recumbent position on the back for blood pressure readings. The mean value of three repeated measurements over a 5-min period was considered.

### Biochemical parameters

Regarding lipid profile parameters, blood samples were drawn from each patient 12 h after an overnight fast. The determination of TC, HDL, and LDL concentrations were performed by direct enzymatic colorimetric methods using “Spinreact-Spain” reagents. However, the glycated hemoglobin (HbA1c) concentration values were determined using an ion exchange resin separation method. For Apo A1 and B, turbidimetric tests (Spinreact Reagents, Spain) were used.

### Nutrient intake assessment

Through a 3-days (2 days of week and a weekend day) food record, we assessed all food and drink intakes. All patients were given strict instructions on how to fill out their questionnaire and how to record all information about type of food, time of the meal, serving size, culinary method, and further details. For patients who were illiterates and/or unable to remember their food consumption, one of their family member was assigned to fill out the food record for them.

### Statistical analysis

The software program NutriSurvey for windows 2007 [17], SEAMEO-TROPMED RCCN-University of Indonesia (NutriSurvey, 2007) was used to calculate the total energy intake, diet composition and nutrients (vitamins, minerals and amino acids) consumption. This program is based on the German food database (BLS) with English names.

All statistical analysis was processed using SPSS 22.0 (Statistical Package for the Social Sciences, IBM Corporation; Chicago, IL, August 2013). Results are expressed as means ± standard deviations, percentages and odd ratios.

Independent Student's *t*-test was used for comparing continuous variables. The χ^2^ test was used for categorical (percentage values) variables comparison. The independent relationships between antioxidants (vitamins and minerals) intake and atherogenic indices (TC/HDL and Apo B/Apo A1 ratios), along with the independent variable, diabetic status, were assessed by multivariate logistic regression models. A *p*-value lower than 0.05 was considered statistically significant with a confidence interval (CI) of 95%.

### Ethical consideration

The study protocol was approved by the scientific and ethical committee of Djillali LIABES University and the director of Health and Population of the *Wilaya* of Sidi-Bel-Abbes, Algeria (agreement No. 142 dated 13 February 2013). All participants gave written approval after the study protocol has been explicated to them.

## Results

The basic characteristics (anthropometric, biochemical parameters and antioxidant intake) of the study population are shown on Table 1. Ninety-five (95) women with type 2 diabetes (T2D) were compared to 93 non-diabetic ones. Comparison of patients with T2D vs. non-diabetic ones reveals statistical differences (*p* < 0.05) for body weight, height, BMI, HbA1c, and total cholesterol levels. Furthermore, significant differences were noted for the TC/HDL ratio, vitamin C, E, and copper dietary intakes.

**Table 1:** Basic characteristics of participants.

**Variables**	**All patients(Mean ± S.D.)**	**Non-diabetic patients(Mean ± S.D.)**	**Type 2 diabetic patients(Mean ± S.D.)**	*p*-value for statistical test^*^
Size, n (%)	188 (100)	93 (49.46)	95 (50.54)	0.859
Age (years)	55.67 ± 12.31	54.07 ± 12.30	55.70 ± 11.16	0.056
Weight (kg)	76.17 ± 12.72	89.53 ± 8.94	73.84 ± 11.82	**<0.001**
Height (cm)	162.08 ± 7.17	168.78 ± 5.43	160.91 ± 6.80	**<0.001**
Waist circumference (cm)	97.84 ± 13.18	98.16 ± 5.12	97.78 ± 14.14	0.890
BMI (kg/m^2^)	28.92 ± 4.35	31.41 ± 2.85	28.49 ± 4.43	**0.001**
Systolic pressure (mmHg)	128.84 ± 14.31	127.57 ± 11.00	129.06 ± 14.83	0.612
Diastolic pressure (mmHg)	76.11 ± 9.80	76.57 ± 9.62	76.03 ± 9.86	0.789
HbA1c (mmol/mol)	58 ± 7.58	45 ± 3.92	61 ± 10.29	**<0.001**
TC (mg/dl)	164 ± 36	136 ± 31	170 ± 35	**<0.001**
LDL-c (mg/dl)	108 ± 31	115 ± 29	107 ± 32	0.206
HDL-c (mg/dl)	39 ± 10	35 ± 4	40 ± 11	0.057
TC/HDL-c	4.42 ± 1.42	3.87 ± 0.96	4.52 ± 1.47	**0.026**
Apo B/Apo A1	0.76 ± 0.33	0.70 ± 0.17	0.77 ± 0.15	0.302
Vitamin A intake (μg)	1533.63 ± 567.23	1362.67 ± 474.90	1563.55 ± 577.98	0.084
Vitamin C intake (mg)	139.50 ± 69.46	106.77 ± 41.21	145.23 ± 71.86	**0.007**
Vitamin E intake (mg)	18.07 ± 6.88	14.93 ± 6.19	18.62 ± 6.86	**0.009**
Zinc intake (mg)	11.82 ± 4.21	12.66 ± 4.95	11.67 ± 4.07	0.253
Manganese intake (mg)	3.03 ± 1.30	2.68 ± 1.20	3.09 ± 1.31	0.131
Copper intake (mg)	2.19 ± 0.40	1.93 ± 0.45	2.24 ± 0.37	**<0.001**

* *Comparison between diabetic and non-diabetic groups, mean values were compared using Student t-test and percentages were compared with Chi-square test, p < 0.05 was considered to be significant; S.D., standard deviation; BMI, body mass index; HbA1c, glycosylated hemoglobin; F TC, fasting total cholesterol; F HDL-c, fasting high-density lipoprotein cholesterol; apo, apolipoprotein. Bold indicates significant p-values*.

The multivariate regression of antioxidant intake quartiles and prediction of atherosclerosis in diabetic and non-diabetic patients, according to the TC/HDL ratio, resulted in non-significant association between antioxidants (vitamin A, Zn, Mg, and Cu) and the atherogenic risk (Table 2).

**Table 2:** Antioxidants intake and prediction of atherosclerosis in diabetic and non-diabetic patients using a multivariate regression model based on TC/HDL-c ratio.

	**All patients odds ratio (95% CI)**	^*^p-value	**Non-diabetic patients odds ratio (95% CI)**	^*^p-value	**Type 2 diabetic patients odds ratio (95% CI)**	^*^p-value
**VITAMINS INTAKE**						
**Vitamin A (μg)**						
Q1 (549–1,052)	Referent		Referent		Referent	
Q2 (1053–1533)	0.380[0.049–2.965]	0.356	0.382 [0.023–2.258]	0.500	0.500 [0.040–3.176]	0.589
Q3 (1534–1883)	0.990[0.101–4.680]	0.993	1.778 [0.081–3.121]	0.715	1.613 [0.133–5.604]	0.707
Q4 (1884–3423)	0.295[0.067–1.798]	0.954	1.261 [0.051–3.262]	0.888	1.470 [0.099–2.916]	0.582
**Vitamin C (mg)**						
Q1 (62–108)	Referent		Referent		Referent	
Q2 (109–121)	1.907[1.671–2.237]	**0.001**	1.68 [1.079–4.203]	**0.007**	2.116 [1.361–3.162]	**0.031**
Q3 (122–153)	3.931[1.183–8.576]	**0.039**	2.502 [0.648–3.447]	0.081	2.420 [0.303–3.127]	0.201
Q4 (154–363)	3.519[2.405–4.408]	**0.009**	3.984 [1.775–7.412]	**0.020**	2.491 [0.343–4.884]	0.213
**Vitamin E (mg)**						
Q1 (3–14)	Referent		Referent		Referent	
Q2 (15–19)	1.101 [0.182–6.684]	0.916	1.650 [0.199–3.679]	0.643	0.768 [0.092–6.421]	0.807
Q3 (20–24)	3.849 [0.772–4.182]	0.100	3.526 [1.097–5.656]	**0.040**	1.653 [0.188–4.519]	0.650
Q4 (25–35)	2.425[2.017–5.715]	**0.012**	1.843 [1.877–2.731]	**0.019**	1.332 [0.682–2.685]	0.094
**MINERALS INTAKE**						
**Zinc (mg)**						
Q1 (6–9.30)	Referent		Referent		Referent	
Q2 (9.40–10.50)	1.188[0.305–4.631]	0.803	1.535 [0.324–7.278]	0.590	1.159 [0.204–6.604]	0.868
Q3 (10.60–12.80)	0.498 [0.099–2.505]	0.398	0.447 [0.059–3.370]	0.435	1.544 [0.141–6.844]	0.722
Q4 (12.90–22.20)	3.168 [0.569–6.625]	0.188	2.604 [0.538–6.422]	0.163	3.488 [0.264–6.144]	0.343
**Magnesium (mg)**						
Q1 (1–2.20)	Referent		Referent		Referent	
Q2 (2.30–2.80)	0.589 [0.187–1.860]	0.367	0.534 [0.146–1.949]	0.342	0.469 [0.141–1.565]	0.218
Q3 (2.90–3.50)	0.638 [0.233–1.747]	0.382	0.932 [0.284–3.061]	0.908	0.433 [0.137–1.372]	0.155
Q4 (3.60–9.30)	0.994 [0.292–3.382]	0.993	1.103 [0.284–4.291]	0.887	0.868 [0.206–3.653]	0.847
**Copper (mg)**						
Q1 (1.20–2.10)	Referent		Referent		Referent	
Q2 (2.15–2.30)	1.217 [0.410–3.613]	0.723	1.258 [0.345–4.590]	0.729	1.238 [0.391–3.924]	0.716
Q3 (2.35–2.40)	0.525 [0.181–1.525]	0.236	0.483-[0.138–1.685]	0.254	0.635 [0.201–1.999]	0.437
Q4 (2.50–3.40)	2.098 [0.663–6.641]	0.208	2.132 [0.567–8.010]	0.262	2.571 [0.713–9.270]	0.149

* *Multivariate logistic regression significant at p = 0.05; CI, confidence interval; Q, quartiles. Bold indicates significant p-values*.

For the whole study population, significant associations were observed regarding vitamin C intake beyond the second quartile with the 2nd, the 3rd, and the 4th quartiles odds ratios of 1.907[1.671–2.237], 3.931[1.183–8.576] and 3.519[2.405–4.408], respectively. In the non-diabetic group, significant relationships were noticed in the 2nd quartile; 1.680[1.079–4.203] and the 4th one; 3.984[1.775–7.412]. However, in patients with T2D, the atherogenic risk was associated with the 2nd quartile of vitamin C intake; 2.116[1.361–3.162], this quartile is over the daily allowance of vitamin C intake (75 mg/d).

Regarding the vitamin E intake, the atherogenic risk in non-diabetic patients was associated with the 3rd and the 4th quartile with odd ratios values of 3.526[1.097–5.656] and 1.843[1.877–2.731], respectively. Among all participants, the highest quartile, almost twice the recommended daily intake of 15 mg/day of vitamin E intake, was more associated with the atherogenic risk with an odd ratio of 2.425[2.017–5.715].

The Apo B/Apo A1 ratio, as a marker of inflammation, seemed more influenced by minerals intake than vitamins as shown on Table 3. The highest quartile of Zn intake (almost twice the recommended daily intake of 8 mg), in non-diabetic patients, was significantly associated to the Apo B/Apo A1 ratio (OR: 0.059[0.006–0.572]).

**Table 3:** Antioxidants intake and prediction of atherosclerosis in diabetic and non-diabetic patients using a multivariate regression model based on Apo B/Apo A1 ratio.

	**All patients odds ratio (95% CI)**	^*^p-value	**Non-diabetic patients odds ratio (95% CI)**	^*^p-value	**Type 2 diabetic patients odds ratio (95% CI)**	^*^p-value
**VITAMINS INTAKE**						
**Vitamin A (μg)**						
Q1 (549–1052)	Referent		Referent		Referent	
Q2 (1053–1533)	0.949 [0.158–5.684]	0.954	0.160 [0.007–3.896]	0.260	1.218 [0.118–5.536]	0.868
Q3 (1534–1883)	1.113 [0.148–8.382]	0.918	0.497 [0.017–7.871]	0.687	0.959 [0.098–9.348]	0.971
Q4 (1884–3423)	1.722 [0.161–8.431]	0.653	2.136 [0.070–8.229]	0.664	2.109 [0.111–8.124]	0.620
**Vitamin C (mg)**						
Q1 (62–108)	Referent		Referent		Referent	
Q2 (109–121)	1.179 [0.184–7.554]	0.862	0.027 [0.001–1.020]	0.051	1.827 [0.193–7.336]	0.600
Q3 (122–153)	1.299 [0.137–2.347]	0.820	0.022 [0.001–0.880]	**0.043**	2.814 [0.170–4.588]	0.470
Q4 (154–363)	1.788 [0.324–9.852]	0.505	0.156 [0.010–2.430]	0.185	1.935 [0.197–9.017]	0.571
**Vitamin E (mg)**						
Q1 (3–14)	Referent		Referent		Referent	
Q2 (15–19)	0.433 [0.088–2.135]	0.304	0.156 [0.018–1.335]	0.090	0.407 [0.064–2.584]	0.340
Q3 (20–24)	0.840 [0.203–3.486]	0.810	0.391 [0.063–2.412]	0.312	0.525 [0.070–3.944]	0.531
Q4 (25–35)	1.638[0.305–8.804]	0.565	0.067 [0.002–1.937]	0.115	1.903 [0.231–5.657]	0.550
**MINERALS INTAKE**						
**Zinc (mg)**						
Q1 (6–9.30)	Referent		Referent		Referent	
Q2 (9.40–10.50)	1.004 [0.280–3.596]	0.995	0.349 [0.069–1.776]	0.205	1.059 [0.211–5.312]	0.944
Q3 (10.60–12.80)	1.643 [0.362–7.455]	0.520	1.362 [0.149–7.415]	0.784	2.800 [0.296–6.482]	0.369
Q4 (12.90–22.20)	0.779 [0.176–3.446]	0.742	0.059 [0.006–0.572]	**0.015**	1.150 [0.101–3.082]	0.910
**Magnesium (mg)**						
Q1 (1–2.20)	Referent		Referent		Referent	
Q2 (2.30–2.80)	0.782 [0.266–2.301]	0.656	1.538 [0.430–5.495]	0.508	0.744 [0.245–2.260]	0.602
Q3 (2.90–3.50)	0.940 [0.353–2.501]	0.901	1.923 [0.561–6.597]	0.298	0.883 [0.293–2.656]	0.824
Q4 (3.60–9.30)	0.534 [0.165–1.729]	0.295	0.794 [0.206–3.065]	0.738	0.595 [0.152–2.324]	0.455
**Copper (mg)**						
Q1 (1.20–2.10)	Referent		Referent		Referent	
Q2 (2.15–2.30)	0.925 [0.320–2.672]	0.885	0.515 [0.135–1.969]	0.332	0.812 [0.270–2.441]	0.711
Q3 (2.35–2.40)	0.338 [0.121–0.946]	**0.039**	0.166 [0.043–0.642]	**0.009**	0.418 [0.140–1.250]	0.119
Q4 (2.50–3.40)	0.613 [0.206–1.826]	0.380	0.192 [0.048–0.766]	**0.019**	0.607 [0.186–1.977]	0.407

* *Multivariate logistic regression significant at p = 0.05; CI, confidence interval; Q, quartiles. Bold indicates significant p-values*.

We analyzed the collected data to assess the potential interaction between Cu intake and Apo B/Apo A1 ratio. The upper quartile of Cu intake (OR: 0.192[0.048–0.766]) was associated to the Apo B/Apo A1 ratio in the non-diabetic group. Likewise, the 2nd quartile (Two to four times the recommended Cu daily intakes of 0.9 mg) was more associated to the Apo B/Apo A1 ratio; OR: 0.338[0.121–0.946] and 0.166[0.043–0.642] in the whole study population and in the non-diabetic group, respectively.

## Discussion

Atherosclerosis is an inflammatory-degenerative complication characterized by the accumulation of lipids, calcium and other elements in the arterial wall. Numerous risk factors have been associated to its progress including e.g., male gender, age, diabetes, dyslipidemia, high blood pressure, and bad lifestyle habits as smoking and physical inactivity [18]. Though, serum cholesterol and especially LDL levels are frequently used as biochemical markers to predict the onset of the atherogenic disease. Currently, these conventional markers are being replaced by lipid-related ratios such as TC/HDL, TG/HDL, and apolipoprotein ratio essentially represented by Apo B/Apo A1 [14, 19–22].

Several hypotheses explain the occurrence and prevention of atherosclerosis. In living organisms, endogenous antioxidant molecules (e.g., uric acid, glutathione, bilirubin, coenzyme Q, lipoic acid, melatonin) and dietary antioxidants (e.g., vitamins E, A, and C and minerals zinc, magnesium, copper, and selenium) limit the harmful effect of oxidants [23]. However, epidemiological studies evaluating the relationship between micronutrients and atherogenic markers (lipids and lipoproteins ratios) are still scarce.

The present cross-sectional study investigated the association between dietary antioxidants intake (vitamins; A, C, and E and minerals; Mg, Zn, and Cu) and atherogenic indices (TC/HDL and Apo B/Apo A1 ratios) in a sample of middle-aged women according to their diabetic profile.

Preliminary results from this study indicate significant differences regarding body weight, height, BMI, HbA1c and total cholesterol levels when comparing between non-diabetics and type 2 diabetic patients. The study identify the discriminatory power of BMI and its association with T2D although, according to literature, when studying diverse ethnic groups the BMI makes comparisons difficult and limits generalizability [24, 25]. Conclusions from the Uppsala study [26] indicated that high values of BMI was associated with an increased risk of T2D, these observations corroborate with our findings.

We noticed elevated lipid profile and lipoprotein profiles in T2D patients when comparing to their non-diabetic counterparts. Furthermore, our results revealed an increase in TC/HDL and Apo B/Apo A1 ratios in diabetic patients comparing to non-diabetic ones. Numerous studies showed the relationship between altered lipid profiles and T2D, and it was further shown that the dyslipidaemia predisposes the diabetic patients to cardiovascular complications, especially atherosclerosis and coronary heart disease [14, 22, 27–30].

Very few epidemiological studies evaluated associations of vitamins with markers of inflammation and atherosclerosis [31–33]. Our data figured out inconsistent associations between micronutrients intake and TC/HDL as atherogenic indices. The highest quartiles of vitamin C and E intakes were associated with a significant risk of atherosclerosis (TC/HDL > 3.0) in all patients and in the group of non-diabetic patients. Despite that statistics from studies have not conclusively reinforced the hypothesis of established effects of antioxidants on atherosclerosis. Theoretically, antioxidants may prevent atherosclerosis by preventing LDL oxidation. Vitamin E incorporates into the LDL particle and protects LDL from oxidation. Inversely, vitamin C, as water soluble molecule, is not incorporated in LDL particle but it has a protective role against LDL oxidation. Additionally, when vitamin C is consumed with higher doses, it might have deleterious impact on the cardiovascular system through possible protein glycation and stimulated lipid peroxidation [34, 35].

Our findings, about the increased risk of atherosclerosis with the highest quartile of vitamin E intake, were unexpected. Vitamin E may be proatherogenic at higher levels nevertheless it is probable that it has an effect on strengthening the tendency to calcify atheroma that could have a beneficial action stabilizing plaque development [33].

By referring to the Apo B/Apo A1 ratio as potential atherogenic indices, no noteworthy associations were observed regarding vitamins intakes; however, significant risk was associated to the highest quartiles of zinc and copper intakes in non-diabetic women.

The evidence of relationships between the cardiovascular risk including atherosclerosis and dietary zinc intake, provided by prospective cohort studies are complicated by inherent confusing factors as gender, smoking, alcohol consumption, and interactions between zinc and other minerals, such as iron and copper. Vashum et al. [36] reported no significant associations between CVD incidences and the higher zinc and zinc/iron dietary intakes in female patients with T2D. However, Otto et al. revealed that dietary zinc intake from red meat were correlated to CVD risks, in non-diabetic patients, despite no association with total zinc intake [37]. These findings corroborate with our results where the 4th quartile of Zn intake was significantly associated to the atherogenic risk (Apo B/Apo A1 > 0.7) in non-diabetic female patients but not in T2D female ones.

The present study found no significant associations between the atherosclerotic risk and copper dietary intake in all patients and diabetic ones; nevertheless, the highest quartile of Cu intake was correlated to the risk of atherosclerosis in non-diabetic patients. Similar to our finding, the study carried out in Iran by Shab-Bidar et al. found no significant associations between dietary intake of copper and metabolic syndrome components including diabetes [38]. The same conclusion was reported by Bo et al. [39].

The results of the current investigation showed no significant associations between magnesium dietary intake and both atherogenic indices (TC/HDL and Apo B/Apo A1 ratios) among the studied groups. Actually, the Mg has advantageous therapeutic uses in preventing cardiovascular events. This mineral is involved in several homeostasis functions and is considered as a cornerstone in some crucial physiological, biochemical, and cellular processes regulating cardiovascular function [40, 41]. Though, further experimental, clinical, and well-designed cohort studies are needed in order to determine the interrelationship between dietary Mg intake, serum Mg^2+^ concentration, and cardiovascular disorders, including hypertension, atherosclerosis, and cardiac arrhythmias.

This study has concerned women of a very sensitive age group, characterized by several metabolic changes expected to accelerate atherosclerosis. The strengths of our study are based on the use of lipid and lipoproteins ratios (TC/HDL and Apo B/Apo A1) as atherogenic indices instead of individual lipid parameters concurrent with a rigorous assessment of antioxidants dietary intakes. In addition, the use of observational methods instead of interventional method provides more information about the eating habits and nutritional status of free-living situation.

The present study is not without limitations. Cross-sectional data do not allow understating causal interactions, and spurious outcomes may occur. The assessment of these associations is indeed difficult due to the complexity of the connection between components of dietary intake and physiological body reactions. Furthermore, the assessment of dietary intakes obtained by a 3-days self-reported record may be influenced by a number of reporting biases. Another limitation of the study is that evidences on the association between antioxidant dietary intake and sera risk indices of atherosclerosis is obtained from intervention studies, while our investigation is purely observational. Reliable information on the CVD mortality in the studied area are not available. A final worth mentioning limitation of the study is the inability to quantify Selenium (Se) dietary intake, through the employed software program (NutriSurvey), which constitutes an important antioxidant that could provide much more clarifications on the relationship between dietary antioxidants and the atherosclerosis risk.

## Conclusion

In conclusion, this study adds weight to the hypothesis aiming to understand atherosclerosis risk in association to the components of antioxidant dietary intakes in women gender. The relationship between dietary antioxidants and atherogenic indices was not necessarily influenced by T2D. Most findings of this study showed that the TC/HDL ratio was more likely to be associated to vitamins antioxidant intake especially vitamin C and E. However, the Apo B/Apo A1 ratio was influenced by the mineral profile (Zn and Cu). Despite that our work, among those of the few other prospective investigations, seems promising, establishing firm conclusions to explore the relationship between cardiovascular disease including atherosclerosis and dietary antioxidants needs further studies to be performed, preferably randomized controlled trials, and large-scale prospective and intervention studies.

## Author contributions

MD: Study design, Collection and assembly of data, Data analysis and interpretation, Final approval of manuscript. MK: Administrative support, Study design, Manuscript writing, Final approval of manuscript.

## Conflict of interest statement

The authors declare that the research was conducted in the absence of any commercial or financial relationships that could be construed as a potential conflict of interest.
